# Computational identification of salient cancer care topics and themes in oncology social work notes

**DOI:** 10.1093/jncics/pkag033

**Published:** 2026-03-30

**Authors:** Ryzen Benson, Swetha Rajkumar, Clodagh Kenny, Michelle Zhao, Ji-Hyun Chang, Theodore Scheel, Lauren Boreta, Julian C Hong

**Affiliations:** Department of Radiation Oncology, University of California San Francisco, San Francisco, CA, United States; Bakar Computational Health Sciences Institute, University of California San Francisco, San Francisco, CA, United States; Bakar Computational Health Sciences Institute, University of California San Francisco, San Francisco, CA, United States; University of California, Berkeley, CA, United States; Bakar Computational Health Sciences Institute, University of California San Francisco, San Francisco, CA, United States; Bakar Computational Health Sciences Institute, University of California San Francisco, San Francisco, CA, United States; Department of Radiation Oncology, University of California San Francisco, San Francisco, CA, United States; Seoul National University Hospital, Seoul, South Korea; UCSF Helen Diller Comprehensive Center, University of California San Francisco, San Francisco, CA, United States; Department of Radiation Oncology, University of California San Francisco, San Francisco, CA, United States; Department of Radiation Oncology, University of California San Francisco, San Francisco, CA, United States; Bakar Computational Health Sciences Institute, University of California San Francisco, San Francisco, CA, United States; UCSF Helen Diller Comprehensive Center, University of California San Francisco, San Francisco, CA, United States

## Abstract

**Background:**

Social work notes provide a multifaceted narrative of a patient’s cancer journey. Topic modeling is a computational technique that can be used to organize notes into thematic clusters and study topics in oncological social work at scale. In this study, we used the BERTopic framework to perform topic modeling of cancer social work notes and identify roles and themes frequently documented in oncological social work.

**Methods:**

We collected 106 676 social work notes from 25 341 patients diagnosed with cancer at the University of California San Francisco. Notes were split at the sentence level, and BERTopic model parameters were optimized experimentally. For each BERTopic cluster, 2 annotators independently assigned labels to 250 randomly selected sentences and keyword sets, with disagreements adjudicated by a third reviewer. Given the same sentences and keywords, 3 large language models independently labelled each cluster. Finally, human-generated and large language model–generated labels were harmonized.

**Results:**

Topic modeling of social work notes revealed 15 topics, classified into 3 distinct subthemes: (1) social worker assessments of patient psychosocial needs and service provision; (2) patient-centric factors, experiences, and the impact of cancer diagnosis on lifestyle; and (3) family and caregiver experiences when caring for someone with cancer.

**Conclusion:**

Topic modeling supplemented by large language model interpretation reveals complex psychosocial themes in patients receiving social work services during cancer care experience. We envision these methods supporting research that clusters oncological social work notes into thematic cohorts for qualitative studies on how social work care differs across patient strata and how topics are differentially distributed across cancer subtypes.

## Introduction

A cancer diagnosis results in profound disruptions and distress in the lives of patients and their support networks.^1-3^ These stressors can be exacerbated in specific patient subpopulations, such as individuals who are uninsured or underinsured or individuals facing housing instability.^4^^,^^5^ Social workers are integral to helping patients navigate their cancer care, performing distress screening, assisting patients with palliative care, and connecting patients with essential resources (eg, lodging and travel arrangements).^6-9^ The services patients receive, however, depend on their cancer subtype, patient-specific factors, and often the context in which social work is administered (eg, academic vs community based).^10^^,^^11^ More research is needed to promptly identify and assess the needs of patients who use social work during their cancer care and to examine how those needs change over time.^12^^,^^13^

Natural language processing techniques have been applied to predict aspects of social service documentation, utilization, and treatment-related decisions based on social needs; however, natural language processing studies in oncological social work are limited.^14-16^ Topic modeling involves clustering text into semantically similar thematic groups and assigning descriptive labels to each group. BERTopic is a modular framework that uses bidirectional encoder representations from transformers (BERT) to embed unstructured text into semantically meaningful word embeddings for informative clustering into topic clusters.^17^ Historically, topic models have relied on manual interpretation of these clusters, which is inherently subjective, prone to bias, and prone to interpretive errors.^18^ Some studies have assessed large language models (LLMs) for topic modeling, but this area remains underexplored.^19-21^ Large language models, especially reasoning models, can process large amounts of input text (eg, OpenAI o3-mini: 200,000 input tokens or approximately 500 pages of text) and may be better than humans at analyzing complex themes at scale, helping mitigate bias and quality concerns seen during manual topic interpretation.^22-25^

The primary objective of this study was to perform topic modeling of oncology social work notes to identify and better understand common themes arising in oncological social work. Our secondary objective was to evaluate how well various LLMs can supplement human-labeled topics and assess whether LLM topic labeling, given the same data, results in higher-quality topic labels and descriptions.

## Methods

### Study setting and data collection

**Figure 1.**
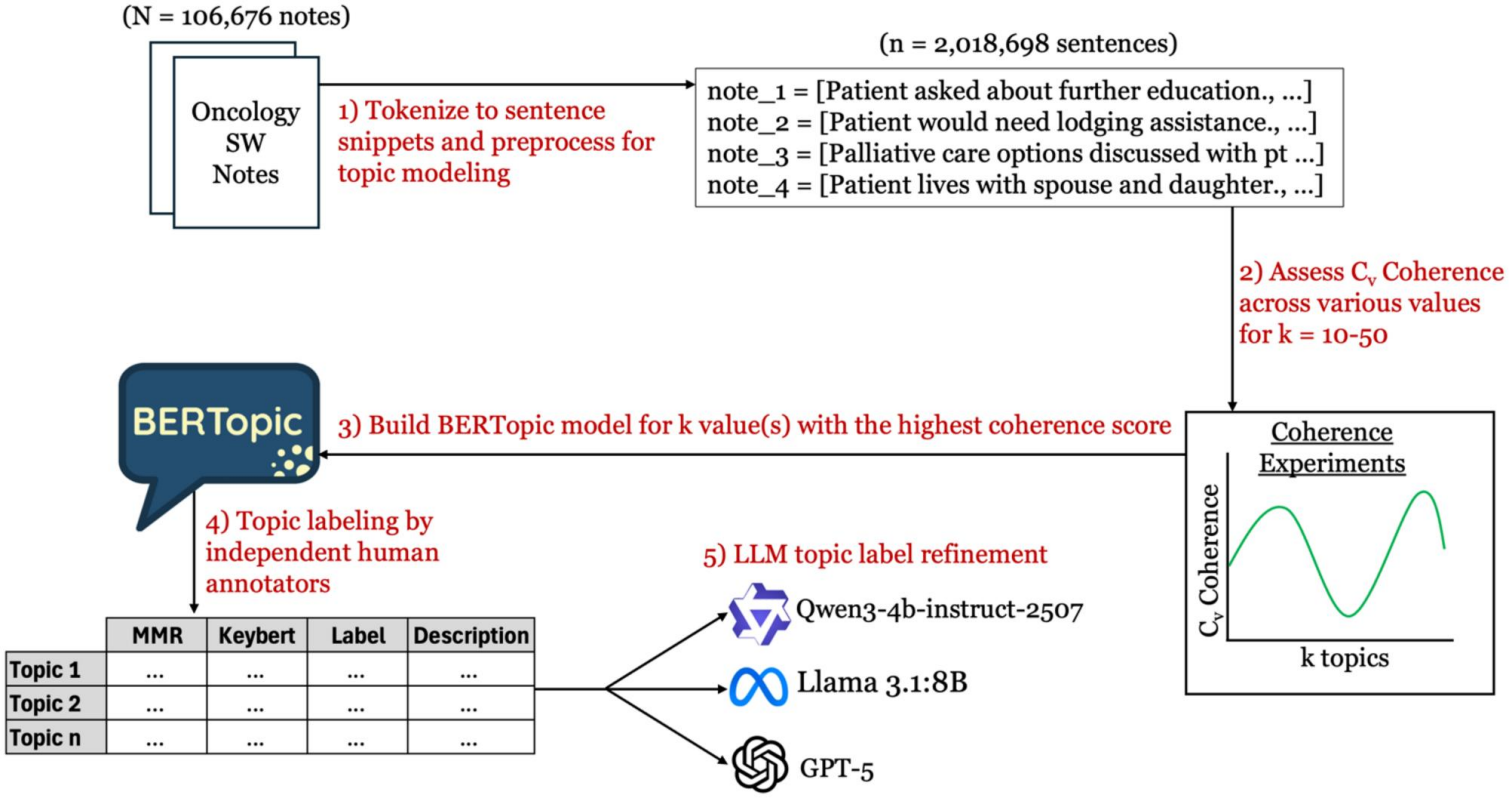
A simplified representation of the study design, consisting of data collection, preprocessing, coherence assessment, BERTopic modeling, and topic labeling. Abbreviation: LLM = large language model.

This study was approved by the University of California San Francisco (UCSF) Institutional Review Board ((# 19-29746)). A simplified overview of the study design is shown in Figure 1. Using *International Classification of Diseases, Tenth Revision, Clinical Modification* codes, we identified 25 341 patients diagnosed with cancer at the UCSF Cancer Center and who saw a social worker following their diagnosis between January 2018 and September 2024.^26^ We restricted the study to patients with at least 2 encounters to exclude individuals with incidental coding errors or individuals diagnosed at UCSF who sought care elsewhere. All social work notes created after the cancer diagnosis date for each patient were collected, resulting in 106 676 notes. Encounter dates were shifted at the patient level to minimize the risk of reidentification and maintain anonymity.^26^^,^^27^

### Data preprocessing

Comorbidities and side effects may manifest following cancer and treatment and can negatively affect patient health and quality of life in the long term.^28^ Therefore, information contained within a single social work note does not exclusively represent a single encounter but often reflects a multifaceted history with complex clinical events, psychosocial factors, and temporal relationships. To better model potentially multiple themes in an encounter, we split each note at the sentence level (2 018 698 sentences). We found common templated phrases, note section headers, and clinical credentials (eg, master of social work, doctor of medicine, registered nurse) that we had to remove before splitting to avoid erroneous splitting errors and incorrect clustering. To identify and remove these values, during model development, we developed regular expressions to flag and remove such instances from the text. Following this text standardization, we then split the note at periods followed by a space (ie, “. ”), resulting in approximate sentence-level snippets. Sentences were embedded using the Alibaba general text embedding model, followed by dimensionality reduction performed using principal component analysis, followed by L2-normalization, and stop word removal (eg, “the,” “a,” “an”).^29^^,^^30^

### BERTopic development and human topic interpretation

Using k-means as our clustering algorithm, we conducted experiments to assess topic coherence (ie, a measure of semantic similarity of text in a cluster) for each of k = 10 to 50 topics. We developed 40 separate topic models, and C_v_ coherence (ie, coherence based on word co-occurrence and topic importance over a sliding window) was evaluated for each.^31^ The models with the highest resulting C_v_ coherence scores were then assessed in a final model, in which the resulting topics would be further labeled and interpreted. In addition to clustering the social work notes, the resulting models were fine-tuned to yield diverse, potentially informative keyword sets to support topic interpretation using the KeyBERT and maximal marginal relevance algorithms.^32^^,^^33^

Once the final BERTopic model was developed, 2 annotators (S.R. and C.K.) with expertise in clinical informatics independently reviewed 250 random sentences, along with maximal marginal relevance and KeyBERT keyword sets, for each cluster of social work sentences. The annotators were then tasked with determining an overarching label that encompassed the sample sentences and keyword sets, along with a description of why that label was chosen. The annotators were tasked with agreeing on a harmonized topic label by discussing their labels and descriptions together, with an independent third-party adjudicator (R.B.) resolving any discrepancies in label designations and guiding the discussion. These harmonized and human-derived labels were the base topic labels to be inductively improved upon following LLM topic interpretation.

### LLM-enhanced topic refinement

**Figure 2.**
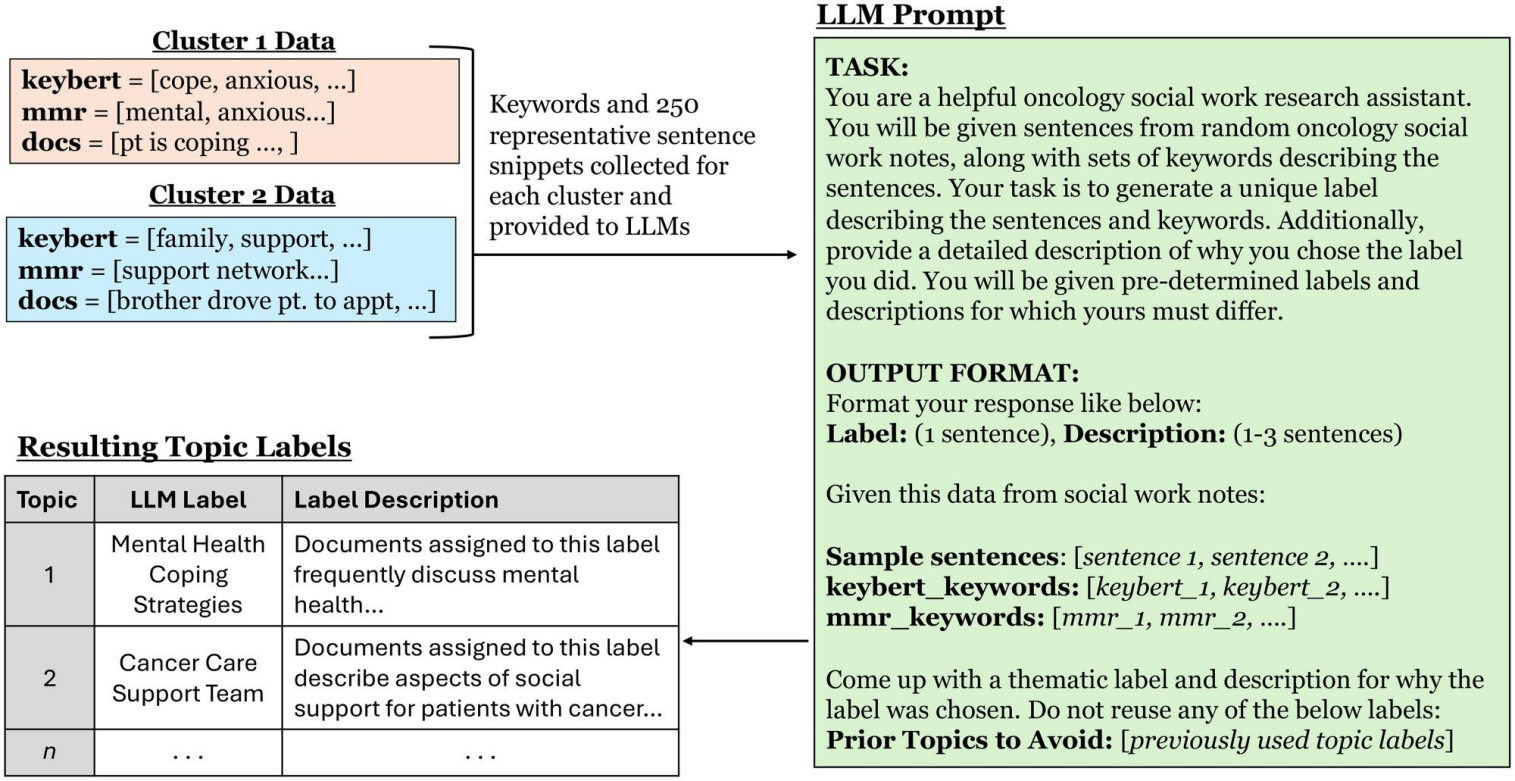
Synthetic example of topic interpretation by large language models (LLMs) using sample sentences and representative keyword sets.

Human interpretation of topic models is often subjective due to personal biases and the difficulty of synthesizing large swaths of complex information. In an attempt to supplement our human-derived labels, we evaluated the topic labeling performance of 3 LLMs: OpenAI’s GPT-5 with high reasoning effort, Alibaba’s qwen3-4b-instruct-2507 (quantization: Q8_0), and Meta’s Llama 3.1:8B (quantization: Q4_K_M).^34-36^ We settled on these models for their demonstrated, reliable performance across various natural language processing tasks and their relatively low computational footprint, enabling widespread and local use on modest consumer hardware. The same random sentences and keyword sets were provided to each model, which was then prompted to generate encompassing topic labels and descriptions for each cluster (Figure 2). GPT-5 was securely hosted through a Health Insurance Portability and Accountability Act–compliant Microsoft Azure deployment using application programming interface (API) version “2025-04-01-preview” and model version “gpt-5 to 2025-08-07.”

## Results

Of the 25 341 unique patients included in our analysis, patients’ mean (SD) age at their initial cancer diagnosis was 57.7 (19.9) years. Patients had an average (SD) 4.2 (8.2) social work encounters, with patients aged 65 and older accounting for 42.3% of all social work encounters, followed by patients aged 45 to 64 years (33.2%), 19 to 44 years (15.0%), and younger than 18 years (6.7%). We observed over time decreases in the proportions of patients younger than 18 years of age and patients aged 45 to 64 years with a social work encounter. Social work encounter rates among patients aged 19 to 45 years remained relatively steady, whereas rates among patients aged 65 years and older increased over time. The majority of patients (*n* = 24 179 [95%]) had their insurance status documented at the time of their cancer diagnosis, with most of these patients having Medicare (*n* = 9717 [40%]), followed by private insurance (*n* = 7256 [30%]), Medicaid (*n* = 6393 [26%]), uninsured/self-pay (*n* = 455 [2%]), and other insurance (*n* = 358 [2%]). Additional demographic data are shown in Table 1.

**Table 1.** Demographics of patients seen by a social worker and insurance status at the time of cancer diagnosis, stratified by year.

	All years	2018	2019	2020	2021	2022	2023	2024
Social work notes, No. (%)	106 676	9082 (8.5)	13 392 (12.6)	15 811 (62.4)	17 491 (16.4)	21 251 (20)	23 025 (21.6)	6598 (6.2)
Sex, No. (%)								
Male	12 357 (48.8)	1713 (50.5)	1822 (49.2)	1957 (48)	2006 (48.9)	2119 (47.4)	2188 (49.3)	552 (48)
Female	12 946 (51.1)	1682 (49.5)	1879 (50.8)	2122 (52)	2084 (50.8)	2338 (52.4)	2243 (50.6)	598 (52)
Unknown	23 (<1.0)	0 (0)	0 (0)	0 (0)	10 (<1.0)	9 (<1.0)	3 (<1.0)	1 (<1.0)
Age, No. (%)								
≤18 y	1692 (6.7)	333 (9.8)	274 (7.4)	248 (6.1)	267 (6.5)	262 (5.9)	250 (5.6)	58 (5.0)
19-45 y	3809 (15.0)	548 (16.1)	542 (14.6)	581 (14.2)	641 (15.6)	688 (15.4)	638 (14.4)	171 (14.9)
46-64 y	8404 (33.2)	1215 (35.8)	1306 (35.3)	1409 (34.5)	1356 (33.1)	1431 (32)	1375 (31)	312 (27.1)
≥65 y	10 701 (42.3)	1299 (38.3)	1579 (42.7)	1841 (45.1)	1836 (44.8)	2085 (46.7)	2171 (49)	610 (53)
Unknown	720 (2.8)	0 (0)	0 (0)	0 (0)	0 (0)	0 (0)	0 (0)	0 (0)
Race, No. (%)								
Asian	3812 (15.1)	463 (13.6)	518 (14.0)	613 (15.0)	665 (16.2)	696 (15.6)	675 (15.2)	182 (15.8)
Black/African American	1622 (6.4)	250 (7.4)	228 (6.2)	256 (6.3)	259 (6.3)	276 (6.2)	286 (6.5)	67 (5.8)
Native American or Alaska Native	164 (<1.0)	23 (<1.0)	20 (<1.0)	32 (<1.0)	25 (<1.0	30 (<1.0)	25 (<1.0)	<20 (<1.0)
Native Hawaiian/Other Pacific Islander	192 (<1.0)	25 (<1.0)	30 (<1.0)	35 (<1.0)	24 (<1.0)	27 (<1.0)	30 (<1.0)	<20 (<1.0)
White	14 110 (55.7)	1870 (55.1)	2112 (57.1)	2348 (57.6)	2304 (56.2)	2507 (56.1)	2368 (53.4)	601 (52.2)
Multiracial	806 (3.2)	119 (3.5)	126 (3.4)	126 (3.1)	119 (2.9)	109 (2.4)	158 (3.6)	49 (4.3)
Other/unknown/declined to respond	4620 (18.2)	645 (19.0)	667 (18.0)	669 (16.4)	694 (16.9)	821 (18.4)	892 (20.1)	232 (20.2)
Ethnicity, No. (%)								
Non-Hispanic or Latino	19 998 (79.0)	2669 (78.6)	2930 (79.2)	3272 (80.2)	3255 (79.4)	3517 (78.8)	3441 (77.6)	914 (79.4)
Hispanic/Latino	4487 (17.8)	633 (18.6)	666 (18.0)	702 (17.2)	705 (17.2)	778 (17.4)	817 (18.4)	186 (16.2)
Unknown/declined to respond	841 (3.3)	93 (2.7)	105 (2.8)	105 (2.6)	140 (3.4)	171 (3.8)	176 (4.0)	51 (4.4.)
Insurance status, No. (%)								
Private	7256 (30)	744 (2.9)	660 (27.7)	806 (29.9)	1038 (30.1)	1816 (31.7)	1743 (29.6)	449 (28.8)
Medicare	9717 (40.2)	900 (36.2)	985 (41.4)	1081 (40.2)	1330 (38.6)	2280 (39.8)	2450 (41.7)	691 (44.4)
Medicaid	6393 (26.4)	775 (31.1)	671 (28.2)	738 (27.4)	949 (27.5)	1447 (25.2)	1457 (24.8)	356 (22.9)
Uninsured/self-pay	455 (1.9)	51 (2.0)	37 (1.6)	48 (1.8)	72 (2.1)	98 (1.7)	116 (2.0)	33 (2.1)
Other/unknown	358 (1.5)	18 (<1.0)	28 (1.2)	19 (<1.0)	57 (1.7)	93 (1.6)	115 (2.0)	28 (1.8)

### BERTopic and LLM topic labeling results

**Figure 3.**
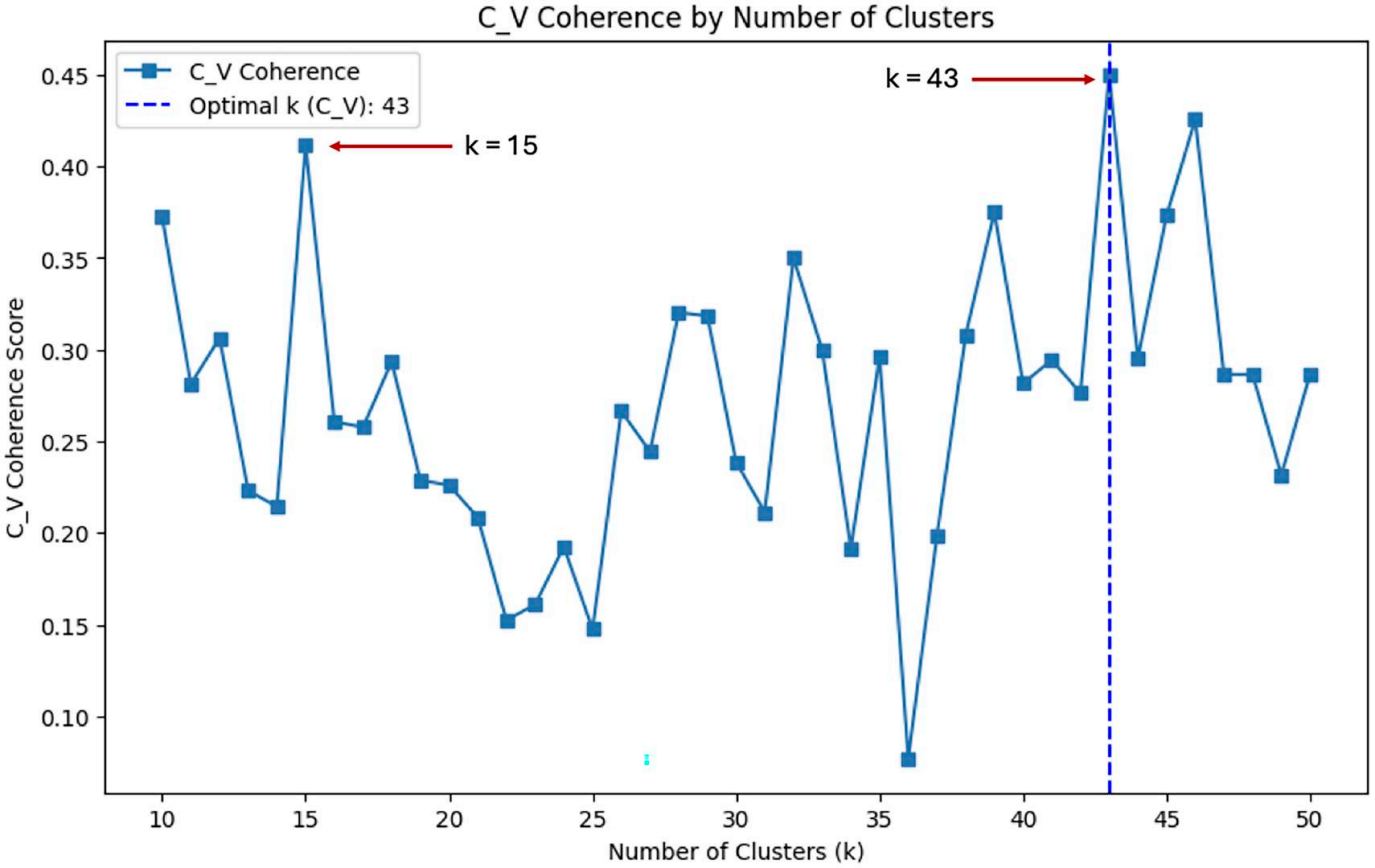
Results from C_V Coherence on models ranging in k from 10 to 50. Models at k = 15 and k = 43 were used in subsequent analyses.

Models at k = 43 showed the highest C_v_ coherence, followed closely by k = 46, and then by k = 15 (Figure 3**)**. Overlapping or redundant themes in the 43 and 46 models, however, suggested potential topic oversaturation. At k = 15, the model topics lacked substantial thematic overlap, covered broad aspects of social work, and were therefore used for our topic labeling experiments. The quality of topic labels and descriptions generated by qwen3-4b-instruct-2507 and GPT-5 was of noticeably higher quality than those generated by Llama 3.1:8B. Both qwen3-4b-instruct-2507 and GPT-5 consistently referenced the keyword sets in addition to the sentence snippets for each cluster. In contrast, Llama 3.1:8B did not appear to use the keyword sets for any topic label.

### Human-derived and LLM-derived topic labels

**Figure 4.**
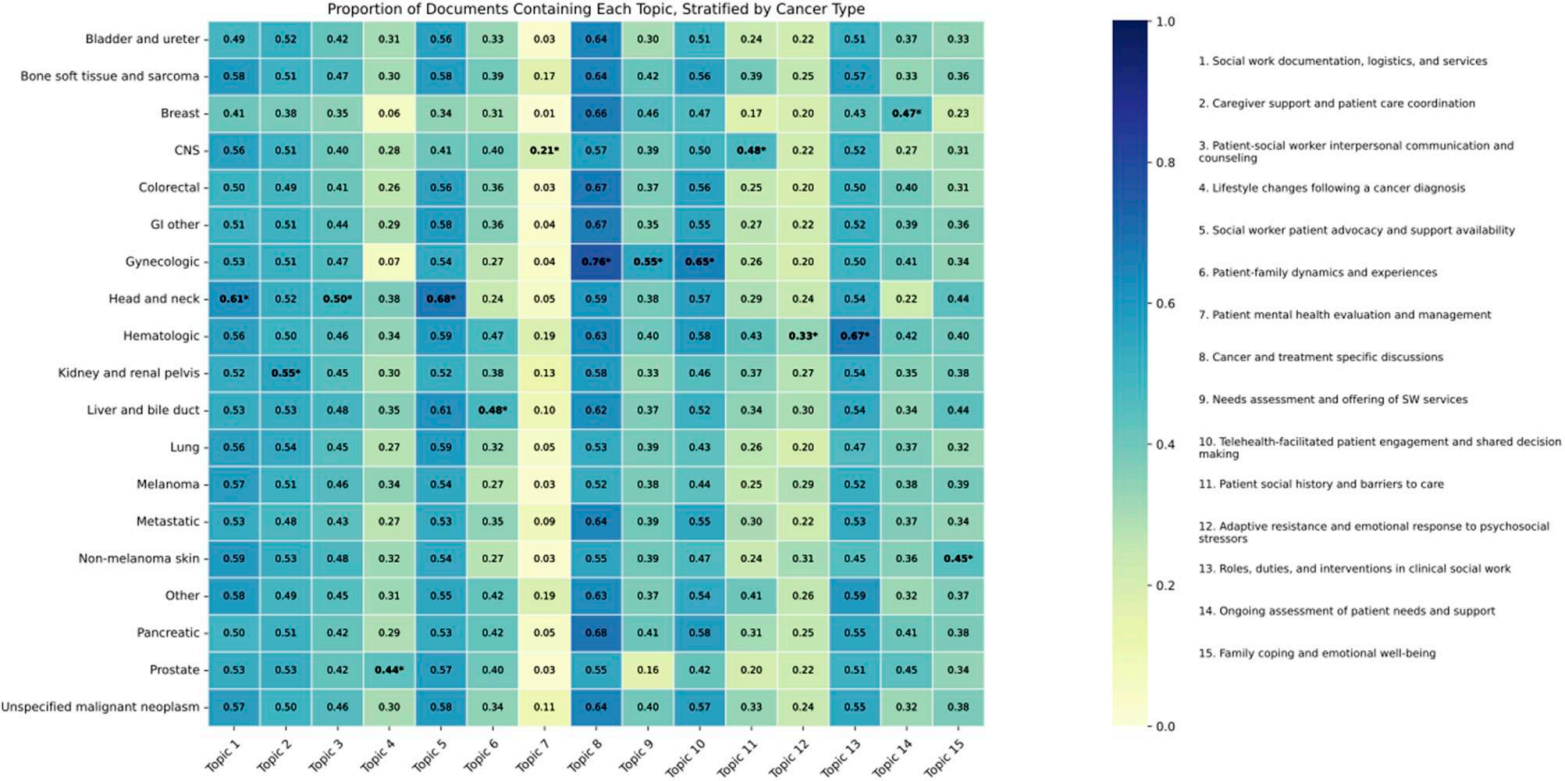
Heat map visualizing the proportion of documents containing each topic, stratified by cancer type. Each topic’s most prevalent cancer type is bolded and denoted by an asterisk.

The final human-labeled and LLM-refined topic labels are shown in Table 2, along with the maximal marginal relevance and KeyBERT keyword sets used to interpret the topics. Figure 4 shows a heatmap of the proportion of social work notes containing each topic subset by cancer type.

**Table 2.** Human-derived and LLM-derived topic labels, along with top KeyBERT and maximal marginal relevance keywords.

Human-LLM topic label	Unique KeyBERT and maximal marginal relevance keywords
Topic 1: Social work documentation, logistics, and services	[‘details recommendations’, ‘additional details’, ‘documentation details’, ‘documentation’, ‘documentation additional’, ‘recommendations contact’, ‘recommendations’, ‘details’, ‘additional’, ‘previous documentation’, ‘lodging’, ‘questions concerns’, ‘encounters’, ‘contact’, ‘lyft’, ‘hours’]
Topic 2: Caregiver support and patient care coordination	[‘caregiver support’, ‘caregivers’, ‘caregiver’, ‘patient information’, ‘patients’, ‘patient’, ‘care coordination’, ‘assistance’, ‘caregiving’, ‘care service’, ‘services’, ‘palliative care’, ‘referral’, ‘medicare’, ‘plan’, ‘interventions’, ‘home care’]
Topic 3: Patient–social worker interpersonal communication and counseling	[‘plan sw’, ‘provided pt’, ‘patient sw’, ‘pt contact’, ‘sw contacted’, ‘sw pt’, ‘sw contact’, ‘called pt’, ‘pt sw’, ‘encouraged pt’, ‘sw received’, ‘contact information’, ‘medical team’, ‘update multidisciplinary’, ‘multidisciplinary medical’, ‘sw met’, ‘team plan’, ‘information sw’]
Topic 4: Lifestyle changes following a cancer diagnosis	[‘therapist’, ‘hopeful’, ‘coping’, ‘able’, ‘health’, ‘anxiety’, ‘needs’, ‘care’, ‘hopes’, ‘helpful’, ‘shared’, ‘reports’, ‘feels’, ‘expressed’, ‘husband’, ‘pt reports’, ‘pt shared’, ‘having’, ‘pt stated’, ‘pain’]
Topic 5: Social worker patient advocacy and support availability	[‘support sw’, ‘sw remain’, ‘advocacy sw’, ‘sw support’, ‘sw encouraged’, ‘sw continues’, ‘support advocacy’, ‘sw provided’, ‘sw remains’, ‘sw continue’, ‘available support’, ‘remains available’, ‘plan sw’, ‘sw available’, ‘available needs’, ‘provided plan’, ‘sw needs’]
Topic 6: Patient-family dynamics and experiences	[‘resides parents’, ‘family members’, ‘family compositionliving’, ‘family’, ‘parents’, ‘family family’, ‘extended family’, ‘familys’, ‘mother father’, ‘parent’, ‘mom’, ‘sister’, ‘siblings’, ‘maternal’, ‘bedside’, ‘grandmother’]
Topic 7: Patient mental health evaluation and management	[‘symptom scores’, ‘assessed pain’, ‘patient notations’, ‘patient report’, ‘symptom’, ‘scores patient’, ‘symptoms’, ‘dyspnea’, ‘individual patient’, ‘pain’, ‘anxiety’, ‘disorder’, ‘mood’, ‘group’, ‘interventions’, ‘mental health’, ‘therapy’, ‘g oals target’, ‘long term’, ‘moderate’]
Topic 8: Cancer- and treatment-specific discussions	[‘myeloma’, ‘multiple myeloma’, ‘patient’, ‘chart patient’, ‘outpatient hematologybmt’, ‘hematologybmt clinic’, ‘leukemia’, ‘oncology’, ‘transplant’, ‘medical’, ‘female’, ‘cancer’, ‘metastatic’, ‘chemotherapy’, ‘medical history’, ‘tumor’, ‘chart medical’, ‘stem’]
Topic 9: Needs assessment and offering of social work services	[‘pt declined’, ‘support pt’, ‘pt denies’, ‘pt denied’, ‘declined needs’, ‘sw pt’, ‘pt endorsed’, ‘offered additional’, ‘additional support’, ‘declined’, ‘inquired pt’, ‘coping pt’, ‘needs pt’, ‘assessment pt’, ‘needs assessment’, ‘support’]
Topic 10: Telehealth-facilitated patient engagement and shared decision making	[‘performed consultation’, ‘consultation using’, ‘verbal consent’, ‘consultation’, ‘telehealth’, ‘telehealth tools’, ‘patient presented’, ‘realtime telehealth’, ‘patient provided’, ‘agreement patient’, ‘transplant’, ‘prior initiating’, ‘answered questions’, ‘interaction’, ‘patient appears’, ‘positive’]
Topic 11: Patient social history and barriers to care	[‘confidentiality’, ‘participants’, ‘unable control’, ‘conduct’, ‘barriers follow’, ‘obligation’, ‘faces barriers’, ‘legal’, ‘comply’, ‘limited support’, ‘denies’, ‘patient denies’, ‘patient denied’, ‘patient does’, ‘declined’, ‘patientfamily limited’, ‘support benefit’, ‘referrals support’, ‘patients’, ‘substance use’]
Topic 12: Adaptive resistance and emotional response to psychosocial stressors	[‘transplant’, ‘health’, ‘adaptive’, ‘good’, ‘able’, ‘adaptive coping’, ‘surgery’, ‘multiple psychosocial’, ‘benefits’, ‘hes’, ‘shared’, ‘feels’, ‘states’, ‘years’, ‘friends’, ‘pain’, ‘expressed’, ‘working’, ‘brother’]
Topic 13: Roles, duties, and interventions in clinical social work	[‘lcsw care’, ‘lcsw clinical’, ‘social lcsw’, ‘oncology social’, ‘concerns lcsw’, ‘phone lcsw’, ‘social outpatient’, ‘clinical social’, ‘outpatient social’, ‘lcsw’, ‘social work’, ‘work note’, ‘palliative care’, ‘msw clinical’, ‘hematology’, ‘inpatient’, ‘outpatient’]
Topic 14: Ongoing assessment of patient needs and support	[‘consulted medical’, ‘psychosocial support’, ‘provide psychosocial’, ‘hospital social’, ‘psychosocial needs’, ‘medical records’, ‘support patient’, ‘reviewed medical’, ‘ongoing psychosocial’, ‘psychosocial’, ‘support’, ‘emotional’, ‘collaboration treatment’, ‘continue assess’, ‘available patient’, ‘social’, ‘ongoing needs’, ‘social work’]
Topic 15: Family coping and emotional well-being	[‘patientfamily adjusting’, ‘patientfamily receptive’, ‘treatment patientfamily’, ‘patientfamily understands’, ‘admission patientfamily’, ‘intervention patientfamily’, ‘patient family’, ‘hospitalization receptive’, ‘patients family’, ‘patientsfamilies’, ‘service intervention’, ‘appears coping’, ‘diagnosis treatment’, ‘patientfamily benefit’, ‘receptive social’, ‘adjusting hospitalization’]

Abbreviation: LLM = large language model.

Topic 1 (“social work documentation, logistics, and services”) was found in 54% of all social work notes (*n* = 57 639). Content in this cluster was more logistically focused, documenting patient encounters, referrals to support services (eg, food and housing assistance), and helping patients navigate their care. The label assigned to topic 2 was “caregiver support and patient care coordination” and was found in half of all social work notes (*n* = 53 248). Sentences in this topic describe conversations among social workers, patients, and their caregivers about required or expected caretaker duties and support available. This support included discussing care plans following cancer treatment; coordinating with various support services during patient discharge; and discussing palliative, hospice, and end-of-life care, when appropriate. Topic 3, labeled “patient–social worker interpersonal communication and counseling,” was found in 43% of all notes (*n* = 46 710) as well as 50% of all head and neck cancer notes. Sentences in this topic often discuss interpersonal communication between the patient and the social worker; outreach activities performed by social workers; and recurring themes of the patient’s psychological experiences, mental health support, and counseling. Despite being present in only 28% of all social work notes (*n* = 29 880), 44% of prostate cancer notes contained topic 4 (“lifestyle changes following a cancer diagnosis”), a higher proportion than any other cancer subtype. Sentences containing this topic often discuss the social worker’s perceptions and assessments of patients’ psychosocial status, including emotional standing, and how effectively patients can concurrently manage aspects of their life after their cancer diagnosis, such as work and family obligations.

Topic 5 (“social worker patient advocacy and support availability”) was found in more than half (52%) of all social work notes (*n* = 55 507) and was the most common topic in head and neck cancer notes (67%). Notes on this topic repeatedly describe ongoing support and services social workers offered to patients and emphasize that social workers are available to address any needs they may have. Topic 6 (“patient-family dynamics and experiences”) was prevalent in more than one-third of all social work notes (*n* = 40 651 [38%]) and was the most prevalent topic in liver and bile duct cancer notes (48%). In this topic, discussions centered on the interplay among the patient, their family, and their support network, including conversations on how best to support the patient during their cancer journey, assessing family hardships and the impact of the cancer diagnosis on the patient’s family, as well as assessing cultural considerations of the patient. Topic 7 (“patient mental health evaluation and management”) was the least prevalent topic across all social work notes (*n* = 11 743 [11%]). Sentences in this topic tended to focus on approaches for assessing patient mental health (eg, administering the 9-item Patient Health Questionnaire/Generalized Anxiety Disorder-7), establishing care goals and monitoring changes in patient mental health, and promoting peer-to-peer connections with other patients. Topic 8 (“cancer- and treatment-specific discussions”) was the most prevalent topic, found in nearly two-thirds of all social work notes (*n* = 64 794 [61%]). Sentences containing this topic were heavily focused on clinical snapshots related to patient cancer treatments more than the psychosocial effects of cancer treatments on patients. These discussions included critical details on the duration of treatment regimens, including chemotherapy, surgery, and stem cell transplants.

Topic 9 (“needs assessment and offering of social work services”), found in 40% of all social work notes (*n* = 42 248), frequently highlights correspondence between the social worker and the patient regarding current and potential social service needs. A recurring theme in this cluster was the consistent provision of social work services and the patient’s consistent declination, either because they no longer needed assistance or because they denied specific needs. More than half (*n* = 55 141 [52%]) of all social work notes contained topic 10 (“telehealth-facilitated patient engagement and shared decision making”), with a consistent underlying theme focused on social work services and contact facilitated through telehealth technologies such as MyChart and telehealth appointments. Text in this cluster appears to show greater agreement and patient participation during social work visits as well as active shared decision making among the social worker, the patient, and the care team. Similar to topic 9, sentences in topic 11 (“patient social history and barriers to care”) describe frequent declination of services due to patient circumstances. Approximately 34% (*n* = 35 715) of all social work notes contained this topic, which differs from topic 9 because the conversations are more focused on aspects of the patient’s social history (eg, family circumstances, substance use) and offering services accordingly. The second-least prevalent topic across all notes was topic 12 (“adaptive resistance and emotional response to psychosocial stressors”), found in just 27% (*n* = 28 376) of social work notes. Sentences in this topic describe how patients are adapting to their illness and treatment, both emotionally and physically. Specifically, these sentences reflect how patients are navigating the psychosocial stressors associated with their diagnosis (eg, pain, uncertainty).

Topic 13 (“roles, duties, and interventions in clinical social work”) was among the most common topics found in our corpus (*n* = 57 523 [54%]). Excerpts in this topic describe the day-to-day tasks and duties of licensed clinical social workers, including ongoing outreach and support to patients, conducting patient chart review, and consulting with clinical staff throughout the treatment journey. Topic 14 (“ongoing assessment of patient needs and support”) was found in 35% of all social work notes (*n* = 40 057). Much like topic 5, sentences from this topic describe a continuous offering of social work services to patients, emphasizing future engagement, support network participation in care and recovery, and long-term care coordination. Finally, similar to topic 6, text clustered into topic 15 (“family coping and emotional well-being”) similarly deals with coping following a cancer diagnosis but is focused more on how the diagnosis affects the patient’s family and support network as opposed to the patient. This topic was prevalent in 37% (*n* = 38 949) of all social work notes and was the most common topic discussed in social work notes for nonmelanoma skin cancers (45%).

## Discussion

Our computational approach clustered more than 106 000 oncological social work notes into 15 thematic topics spanning all facets of cancer care and shedding light on the critical roles social workers play. We envision these methods supporting future research that clusters oncological social work notes into thematic cohorts for qualitative studies on how social work care differs across patient strata and how these topics are differentially distributed across distinct cancer subtypes.

We observed some thematic overlap across multiple topics (eg, topics 6 and 15 both describe facets of the patient’s family). We also, however, identified key differentiating factors between these overlapping clusters (eg, topic 6 focused more on patient-family dynamics, whereas topic 15 described the emotional burden of cancer on family and caregivers, respectively). As a result, even seemingly similar topics highlight critical aspects of social work across the patient, clinician, and caregiver levels. Topics typically fell into 1 of 3 subthemes—(1) social worker assessments of patient psychosocial needs and provision of services; (2) patient-centric factors, experiences, and the impact of cancer diagnosis on lifestyle; and (3) experiences of family members and caregivers taking care of someone with cancer—with many notes containing an interplay of these themes.

The most common unifying theme in our topic model was the various roles of social workers, including patient outreach and support activities, managing referrals, and assessing the psychosocial effects of treatments and cancer on patients. Patients with differing personal circumstances, treatment preferences, and types of cancer require varying levels of social work intervention, highlighting the importance of screening for patient needs, including distress levels, financial insecurity, and the need for housing and transportation assistance.^37^^,^^38^ Mixed-methods research has shown that despite a majority of patients being satisfied with their care after an advanced cancer diagnosis, nearly one-third of patients still sought more information about their cancer treatment and disease prognosis.^39^ To ensure that patient needs are adequately met, continual social worker outreach and communication with the patient are paramount. Our topic modeling approach provides a population-level view of how social work services are delivered across various cancer types and patient strata. Future research may use this approach to cluster specific patient populations (eg, patients with head and neck cancer) and qualitatively evaluate social work practices across these subgroups to identify opportunities for improvement and practices that may translate well to other cancer types. In addition, text clustered under this topic often describes interactions among social workers, members of the care team, and social service organizations, providing a rich resource for exploring varying workflows for supporting patients.

One of the more critical aspects of cancer care involves social workers screening patients’ needs to better guide their care plans.^40^^,^^41^ This is where the focus of social work documentation shifts from purely noting clinical events and decisions to comprehensively outlining the patient’s social and clinical history and how these characteristics may affect the patient’s cancer care. As a result, effective communication between the social worker and the patient is integral to ensure that patient needs that arise throughout their cancer care are promptly identified and addressed. We observed multiple topics describing frequent discussions between the patient and the social worker. These conversations were vibrant, offering meaningful insights into patient details and characteristics that may inform treatment and social work care decisions. Some patient-centric topics focused more on patients’ social and economic factors. In contrast, others focused more on patients’ emotional well-being and the impact of their cancer on activities of daily living (eg, work, family obligations).

In addition to the themes above, a central component of these notes focused on the experiences of family members and caregivers while caring for someone with cancer. This is particularly true for patients with advanced or burdensome cancers that require increased caretaking and assistance from their support network. Social workers play a critical role in helping support networks prepared to care for patients with cancer, providing education about the disease and its treatments to patients and their support networks, having informed conversations about required or expected caretaker duties, and connecting patients and their families to resources tailored to their needs. Social workers are also tasked, however, with monitoring the emotional toll of a cancer diagnosis on the family; coordinating various support services during patient discharge; and discussing palliative, hospice, and end-of-life care, when appropriate.

Zeng et al.^42^ presented an approach using open-source and proprietary LLMs to generate additional topics from previously identified topics through topic modeling (ie, third-level topic modeling). We describe a similar approach here: performing human-labeled topic modeling to establish base topics, then supplementing them with LLM-derived labels. This was done to identify more complex or subtle themes in social worker notes that a human might have missed. We observed overlap between human-labeled and LLM-labeled topics but found that LLMs can synthesize much more information than humans and generate more informative, descriptive labels, thereby improving the quality of human labels. Future research into social work practice and healthcare service delivery should consider our topic modeling approach to create specialized patient cohorts and identify salient qualitative themes in oncological social work notes.

Our study has some limitations to address. First, topic modeling is typically performed at the document level, where entire notes are clustered into similar thematic clusters. Given the wide-ranging roles of social workers in cancer care, we anticipated that social work notes would likely cover multiple topics per note and decided to perform sentence-level topic modeling to capture additional themes within each note. Although our sentence-level topic modeling did identify multiple topics per social work note on average, miscellaneous text, such as non–social work–related text, filler sentences, and templated phrases found in the electronic health record, is still clustered into topics, regardless of their relevance or importance. Therefore, the actual signal of important topics may be diluted by noninformative sentences. Second, our topic modeling groups all patients into a single model, regardless of cancer type, disease severity, treatment regimen, or personal characteristics. As a result, the true prevalence of the topic cannot be readily ascertained for specific patient subpopulations (eg, patients with advanced disease). Second, LLMs have different architectures, undergo distinct training, and their post-training sets will differ from those of other models. How models arrive at their final answer and the intermediate reasoning processes along the way depend on various factors, including model size and complexity, and constitute an intriguing angle of research. In our study, we restricted our analysis to smaller LLMs that fit on most consumer hardware (including an 8 billion– and a 4 billion–parameter model), inherently limiting the model’s intelligence and lagging the performance of proprietary closed-source models or larger open-source models. Finally, our study was conducted at a single institution (a comprehensive cancer center), with a patient population more diverse than the general US population. As a result, the themes and demographic patterns observed here may be unique to our population, and our findings, at this time, cannot be generalized to patients with cancer seeking social work services in other settings.

Our study also has several notable strengths. Topic modeling evaluations in oncological social work are lacking, and previous studies are limited by size.^14^^,^^15^^,^^43^ This study provides a qualitative overview of themes discussed in oncological social work encounters, helping address this gap. In addition, we used LLMs to enhance the quality of human-generated topic labels, providing an alternative to solely manual topic interpretation while preserving human concordance. Finally, the literature continues to stress the need for and the current lack of the use of real-world data in LLM-focused studies.^44^ This study analyzed more than 100 000 deidentified oncology social work notes, providing an extensive LLM-supported analysis of social work notes using real-world data.

## Conclusion

We demonstrated that topic modeling can be used to cluster oncological social work notes into meaningful and thematically similar groups. Clusters resulting from topic modeling can then be used to study differences in social work services delivery, identify psychosocial themes across cancer subtypes and patient strata, and identify gaps and opportunities for improvement in cancer care.

## Data Availability

The social work note data used in this study are not available for request due to concerns about patient anonymity and the potential for reidentification of protected health information. The code used in this study for coherence experimentation, topic modeling, and LLM topic interpretation (including prompts and sampling parameters), however, is available at the GitHub repository (https://github.com/ryzenbenson/Social_work_topic_modeling).
